# The Sense of the Body in Individuals with Spinal Cord Injury

**DOI:** 10.1371/journal.pone.0050757

**Published:** 2012-11-29

**Authors:** Bigna Lenggenhager, Mariella Pazzaglia, Giorgio Scivoletto, Marco Molinari, Salvatore Maria Aglioti

**Affiliations:** 1 IRCCS Fondazione Santa Lucia, Rome, Italy; 2 University Hospital of Child and Adolescent Psychiatry, University of Bern, Bern, Switzerland; 3 Department of Psychology, University of Rome “La Sapienza”, Rome, Italy; University of Bologna, Italy

## Abstract

Increasing evidence suggests that the basic foundations of the self lie in the brain systems that represent the body. Specific sensorimotor stimulation has been shown to alter the bodily self. However, little is known about how disconnection of the brain from the body affects the phenomenological sense of the body and the self. Spinal cord injury (SCI) patients who exhibit massively reduced somatomotor processes below the lesion in the absence of brain damage are suitable for testing the influence of body signals on two important components of the self–the sense of disembodiment and body ownership. We recruited 30 SCI patients and 16 healthy participants, and evaluated the following parameters: (i) depersonalization symptoms, using the Cambridge Depersonalization Scale (CDS), and (ii) measures of body ownership, as quantified by the rubber hand illusion (RHI) paradigm. We found higher CDS scores in SCI patients, which show increased detachment from their body and internal bodily sensations and decreasing global body ownership with higher lesion level. The RHI paradigm reveals no alterations in the illusory ownership of the hand between SCI patients and controls. Yet, there was no typical proprioceptive drift in SCI patients with intact tactile sensation on the hand, which might be related to cortical reorganization in these patients. These results suggest that disconnection of somatomotor inputs to the brain due to spinal cord lesions resulted in a disturbed sense of an embodied self. Furthermore, plasticity-related cortical changes might influence the dynamics of the bodily self.

## Introduction

The sense of the body, an undeniably important aspect of the self, is a complex process that requires the integration and organization of multiple sensory inputs from the somatomotor, vestibular, exteroceptive, and interoceptive systems. To obtain a stable bodily self, the brain generates moment-to-moment representations by integrating and weighting different sensory inputs according to their reliability [1] and presumably integrating them into offline body representations (see e.g. [2] for an alternative distinction). Under normal circumstances, these different representations are integrated to form a coherent and accurate basis for the sense of one’s body and of the self. However, in various neurologic and psychiatric conditions, as well as during certain experimental conditions, this integration process may fail and produce erroneous and disturbed body percepts (see e.g. [3] for a review). The present study investigated whether the massive alteration of somatic afferences from the body to the brain and of motor efferences from the brain to the body affects the multimodal integration of the remaining sensory inputs with respect to the sense of the body and of the self. Patients with spinal cord injury (SCI) with varying injury severity and lesion height can be an ideal model for testing the influence of somatomotor signals on body ownership, embodiment, and their interrelation. Depending on the level and completeness of the spinal cord lesion, these patients demonstrate more or less pronounced loss of sensory and motor functions. While corresponding brain functions are intact in these patients, the loss of somatomotor information about the body part below the lesion level leads to important structural and functional cortical reorganization (e.g. [4]), particularly in the somatomotor areas representing the body. To date, little is known about how this reorganization changes the phenomenological sense of the body, such as the feeling of being embodied and ownership for one’s own body.

The primary aim of this study was to to describe how the disconnection from bodily somatic, motor, and/or autonomic functions that results from SCI might alter phenomenological aspects of self-consciousness depending on the level and completeness of the lesion. For this purpose, we used a well-validated scale that includes various possible alterations in the self-perception, namely the Cambridge Depersonalization Scale (CDS). Depersonalization and derealization disorder is defined by the ICD-10 as the feeling that one’s own experiences are detached, distant, not one’s own, or somehow lost. Depersonalization has been attributed to a failure of sensory integration into a preexisting body model [5]. This idea was confirmed by a recent brain imaging study that associated depersonalization with functional abnormalities in primary (visual, somatosensory, and auditory), secondary, and multisensory areas and in areas responsible for the integration of one’s body schema [6]. Thus, it is likely that a mismatch between a preexisting body model and actual sensory input caused by a dramatic loss of sensory input(s) could cause depersonalization-related symptoms. This hypothesis has been confirmed by experimental deprivation studies, which showed that extreme reduction of bodily sensory input in healthy participants leads to depersonalization symptoms and other disturbances of self-consciousness (e.g. [7]). In the same line, patients with acute sensory (i.e., vestibular) loss and resulting conflicting visuo-vestibular inputs demonstrate stronger symptoms of depersonalization than do healthy persons [8], [9]. We thus hypothesized that patients with reduced somatosensory input strength due to spinal cord lesions would show stronger mismatch between a preexisting body model and sensory input as well as a stronger inter-sensory (e.g., somatosensory–visual) conflict than healthy participants, leading to elevated depersonalization scores. Accordingly, we also hypothesized that greater disturbance of the self would be associated with increasing conflict, i.e., that there would be a positive relationship between the depersonalization score and the extent of somatomotor functional loss (which in turn would correspond to the spinal height level and completeness of the lesion).

The second aim of the study was to conduct a detailed investigation of changes in body ownership, which comprise one important aspect of bodily self-consciousness; this was accomplished using not only phenomenological but also experimental approaches to quantify objective changes. We used the rubber hand illusion (RHI) paradigm, which has been used extensively in recent years, to manipulate and measure body ownership and investigate the processes that underlie multisensory integration dominance. In this paradigm [10], the patient’s own hand is hidden, and a rubber hand is visible. Synchronous stroking of both the patient’s hidden hand and the visible rubber hand leads to illusory ownership of the latter. It is commonly assumed that this illusion occurs because of visual capture of tactile and proprioceptive information in conflicting multisensory situations, which leads to spatial re-calibration of the location of the touch with respect to the sensed position of the hand (proprioceptive drift). We expected that compared to healthy subjects, patients with SCI would show stronger visual capture, because they have to rely more strongly on visual cues to localize (affected) body parts and are thus forced to base multisensory integration on the more-reliable visual cues. A beautiful narrative description of such dependence of vision for the bodily self in a paraplegic patient can be found in a book by Jonathan Cole [11], who describes for example: “Her ‘sense of touch’ on the skin, which was amazingly vivid, seemed dependent on seeing that touch at a certain place and then elaborating it from a visual to sensory/tactile experience.” Such strong visual capture should result in stronger proprioceptive drift among patients who have reduced tactile and proprioceptive hand sensation (i.e., tetraplegic patients) and in enhancement of illusory body ownership.

This hypothesis is also in line with recent data, which showed bi-directional influence between the RHI and body temperature: body temperature and tactile accuracy are decreased during the RHI [12], and conversely, cooling a limb increases the strength of the RHI [13]. It can thus be assumed that decreased tactile and proprioceptive sensitivity (as occurs in tetraplegic patients) will increase the RHI.

## Methods

### Ethical Statement

Written informed consent was obtained from all the participants. The study protocol was approved by the local ethics committee (IRCCS Ethics Committee at Fondazione Santa Lucia, Rome, Protocol CE/PROG.309-16) and was in accordance with the ethical standards of the Declaration of Helsinki.

### Participants

Thirty SCI patients (4 females, mean age 40.8±2.3 years; Table 1) and 16 healthy subjects (6 females, mean age = 40.3±2.9 years) were examined. Patients consecutively admitted for treatment of complications associated with SCI to the Hospital of the Fondazione Santa Lucia (Rome) were screened for participation in our project. SCI patients were grouped according to the level of spinal cord lesion, resulting in a group of 15 patients with paraplegia, with lumbar or thoracic lesions (mean age, 41.3±2.9 years), and a second group of 15 patients with tetraplegia, with cervical lesions (mean age, 40.2±3.7 years). The SCIs were of traumatic (n = 23) or non-traumatic (n = 7) origin, and the time since the lesion onset ranged from 45 days to 18 years (mean time, 38.0±18.1 months in patients with paraplegia and 27.8±10·5 months in patients with tetraplegia). As for the RHI, it is crucial to feel the touch on the hand, so we excluded from the analysis one subject who was unable to report any tactile sensation on any finger. Moreover, to perform the RHI on homogenous samples, we also grouped the patients independent of anatomical lesion level, according to complete or reduced tactile sensation on the back of the left hand.

**Table 1:** Demographic and clinical data of the individuals with spinal cord injury.

Case	Age	Time since injury (days)	Gender	Lesion level	Etiology	AISgrade	Motor level		Sensory level		Sensitivity
							*Left*	*Right*	*Left*	*Right*	*Left hand*
**P1**	19	310	M	T10	Traumatic	A	T10	T10	T10	T10	100
**P2**	34	66	M	L1	Traumatic	D	L3	L3	L1	L1	100
**P3**	42	760	M	T10	Traumatic	A	T10	T10	T10	T10	100
**P4**	42	970	M	T9	Neoplastic	A	T9	T9	T6	T9	100
**P5**	46	310	M	T7	Traumatic	A	T7	T7	T7	T7	100
**P6**	35	6480	M	T7	Traumatic	A	T7	T7	T7	T7	100
**P7**	56	260	M	L1	Traumatic	C	L1	L1	L3	L3	100
**P8**	35	297	M	T10	Traumatic	A	T11	T11	T11	T11	100
**P9**	49	45	F	T12	Traumatic	A	T12	T12	T12	T12	100
**P10**	34	66	M	L1	Traumatic	A	L1	L1	L3	L3	100
**P11**	42	940	M	T3	Traumatic	A	T6	T6	T6	T6	100
**P12**	43	55	F	T7	Neoplastic	C	L3	L3	T7	T7	100
**P13**	65	458	M	T4	Traumatic	C	T4	T4	T4	T4	100
**P14**	36	130	M	T3	Traumatic	A	T3	T3	T3	T3	100
**P15**	49	6239	F	T7	Neoplastic	D	L3	L3	L1	L1	100
**T1**	45	70	M	C6	Neoplastic	D	+	+	C7	C7	27
**T2**	55	58	M	C6	Traumatic	B	C6	C6	+	+	100
**T3**	38	165	M	C4	Traumatic	A	C4	C4	C4	C4	0
**T4**	30	180	M	C6	Traumatic	A	C6	C6	C6	C6	74
**T5**	31	393	M	C6	Traumatic	B	C6	C7	C6	C6	95
**T6**	39	580	M	C4	Traumatic	A	C5	C5	C4	C4	54
**T7**	46	700	M	C7	Vascular	D	C8	C8	+	+	100
**T8**	19	285	M	C6	Traumatic	B	C5	C5	C7	C7	66
**T9**	27	59	M	C6	Traumatic	B	T1	T1	+	+	100
**T10**	61	605	M	C7	Vascular	D	C7	L3	C7	C7	50
**T11**	71	2160	M	C8	Vascular	D	C8	C8	C8	C8	66
**T12**	29	3600	M	C5	Traumatic	A	C6	C6	+	+	100
**T13**	28	3639	M	C6	Traumatic	C	C5	C5	C8	C8	97
**T14**	35	190	M	C6	Traumatic	A	C6	C6	C6	C6	48
**T15**	41	57	M	C5	Traumatic	C	C8	C8	C5	C5	31

**Figure 1:**
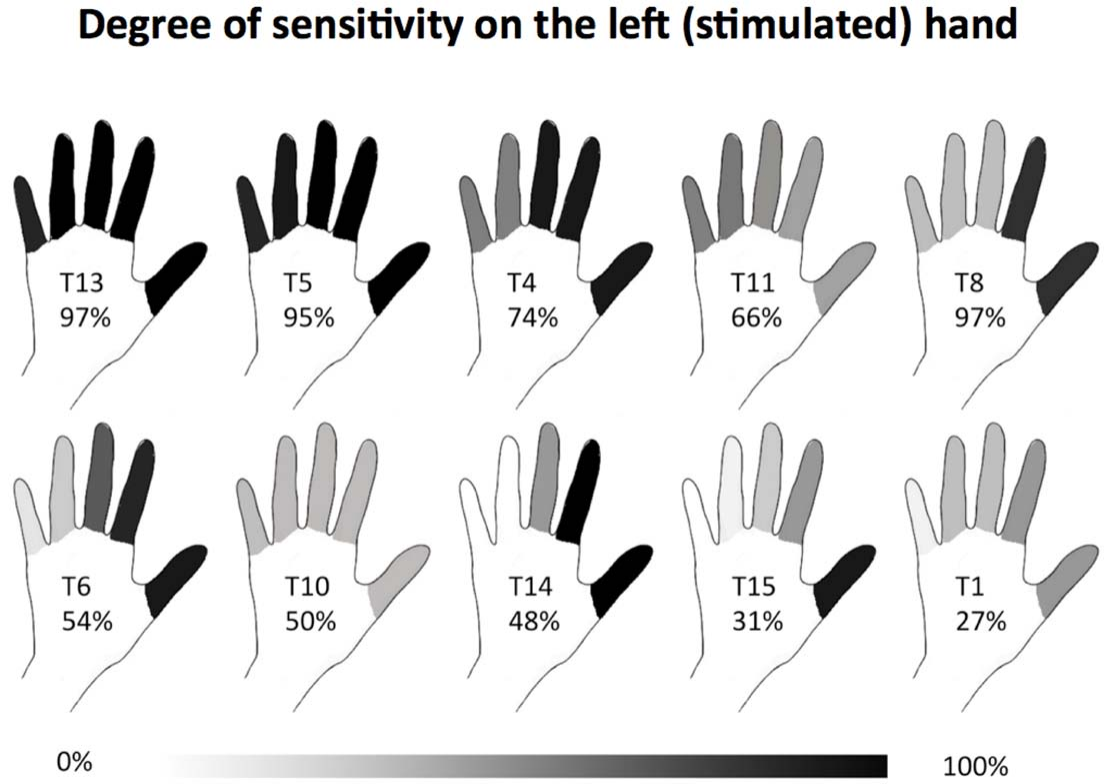
Subjective rating of tactile sensation on the stimulated hand in the S − **group.** Percentages indicate the strength of tactile sensation on each finger compared to the face and is represented as a gray scale from white (no sensation) to black (complete sensation, as on the face). The hands are sorted from the top left to the bottom right by the mean values, which are added to each hand numerically.

To assess the reduced tactile sensation, all of the tetraplegic patients were asked to use a 10-cm visual vertical analogic scale (VAS) to rate tactile sensation strength compared to the sensation of the same stroking on the face. Patients in whom stimuli on the left hand finger were reported as less intense than stimuli on the face were assigned to the S−group (n = 10, see Figure 1). The remaining 19 patients did not show any signs of tactile deficit (S+ group).

Neurologic status was assessed according to the American Spinal Injury Association (ASIA) standards on the basis of the patients’ motor and sensory scores, lesion level, and neurologic impairment. The completeness of the lesion was defined according to the concept of sacral sparing: sensory preservation of the perianal zone and/or motor function of the external anal sphincter (preservation of the lower sacral segments). The lesion was complete in 15 patients (ASIA A) with complete motor and sensory loss below the lesion level and incomplete in 15 patients (ASIA B, C, or D). None of the SCI patients had suffered a concomitant head or brain lesion. All participants had normal or corrected-to-normal vision and no history of neurologic or psychiatric disease.

### Experimental Tasks

Two different tasks, namely the CDS and the RHI paradigm, were used in order to pinpoint changes in the bodily self among the different groups. The sequence of tasks was counterbalanced to exclude any ordering effects.

#### Cambridge Depersonalization Scale

An Italian version of the CDS [14] was given to the participants. This instrument is a well-validated–self-rating scale designed to assess disturbance of the apparent reality of one’s physical state as well as altered perception of bodily experience, symptoms that are thought to characterize depersonalization and derealization disorder [15]. The questionnaire showed high internal consistency (Cronbach’s alpha of 0.89) and good reliability (split-half reliability of 0.92) [14]. The scale comprises 28 items that are rated on 2 separate Likert scales, one for frequency and the other for the duration of the symptoms. In accordance with the suggestions provided by Sierra et al. [16], we calculated the arithmetic sums of the frequency and duration domains to obtain an index of item intensity (range 0–10). This analysis was introduced by the authors to give equal weight to frequent but short-lived and less-frequent but long-lasting depersonalization experiences. A total score of <70 indicates a clinically relevant depersonalization disorder. The scale was adapted in this study by reducing the time period of reference from the original 6 months to 1 month, to ensure that the entire period of reference fell within the time after lesion for all patients (minimum time since lesion was 45 days).

#### Rubber hand illusion procedure

A classical RHI task [10] was administered. Each participant was seated in front of a table. The experimenter placed the participant’s arms in a standard anatomical position inside a wooden box. Both hands were covered with pieces of black cloth so the participant was unable to see them. A life-sized and realistic-looking rubber left hand was placed inside the box in front of the participant and aligned with that participant’s midline. The distance between the participant’s actual left index finger and the index finger of the rubber hand was fixed at 13 cm. The participant’s actual hands remained covered during the stroking procedure (stimulation) and the rubber hand was visible. The participant was asked to maintain visual fixation on the rubber hand and instructed not to move either their hands or head during the test phase. Two identical small paintbrushes were then used to stroke both the rubber hand and the participant’s hidden hand in two blocks either synchronously or asynchronously. The order was alternated between subjects. The brush strokes were applied to the dorsal surface of all fingers for 2 min with an approximate rhythm of 1 brush stroke/s.

#### Proprioceptive drift

Although a number of objective measures of the RHI have been employed in recent years (see e.g. [17] for a review), is thought to be an objective measure of how well a rubber hand is integrated into the participant’s body schema. Successful capture of visual stimuli that convey proprioceptive and tactile cues is thought to bias the apparent location of the arm after synchronous stroking to produce the RHI. To evidence such an effect, immediately after the stimulation, a wooden board with a ruler was inserted into the box covering the participant’s real hands and the rubber hand. The participant was instructed to verbally indicate under which number on the ruler the middle point of their left index finger was located. They were instructed to be as precise as possible and to report the values in centimetres and millimetres. To avoid response bias, the ruler was attached to the board using a hook-and-loop fastener to allow for systematic variance of the offset in each trial. Proprioceptive drift was measured as the difference between the number indicated by the participant and the offset of the ruler measured in centimetres.

#### Subjective ownership

After each block, the participants filled out an Italian version of the 9-item questionnaire regarding their subjective perceptions of illusion during the stimulation [10]. The participants were asked to indicate their level of agreement with each item on a 7-point Likert scale ranging from -3 (‘I totally disagree’) to +3 (‘I totally agree’). The first three questions were designed to capture the experience of the illusion in its two components of referred sensation (Q1 and Q2) and sense of hand ownership (Q3), whereas the other questions were designed to be control questions.

#### Procedure for assessing the proprioceptive baseline

To determine the individual baseline concerning the perceived position of the occluded left index finger, the participants were asked to localize their index finger 5 times prior to start the RHI stimulation. The participants were seated in front of the same wooden box, but in this baseline procedure, the box was always covered with the wooden board. The participants were instructed to close their eyes. They were asked to then open their eyes and indicate under which number on the ruler the middle point of their index finger was located (as described above) and then close their eyes again immediately (see [18] for a similar approach). To eliminate response bias, the offset of the ruler was changed before each of the 5 trials.

## Results

### Cambridge Depersonalization Scale

**Figure 2:**
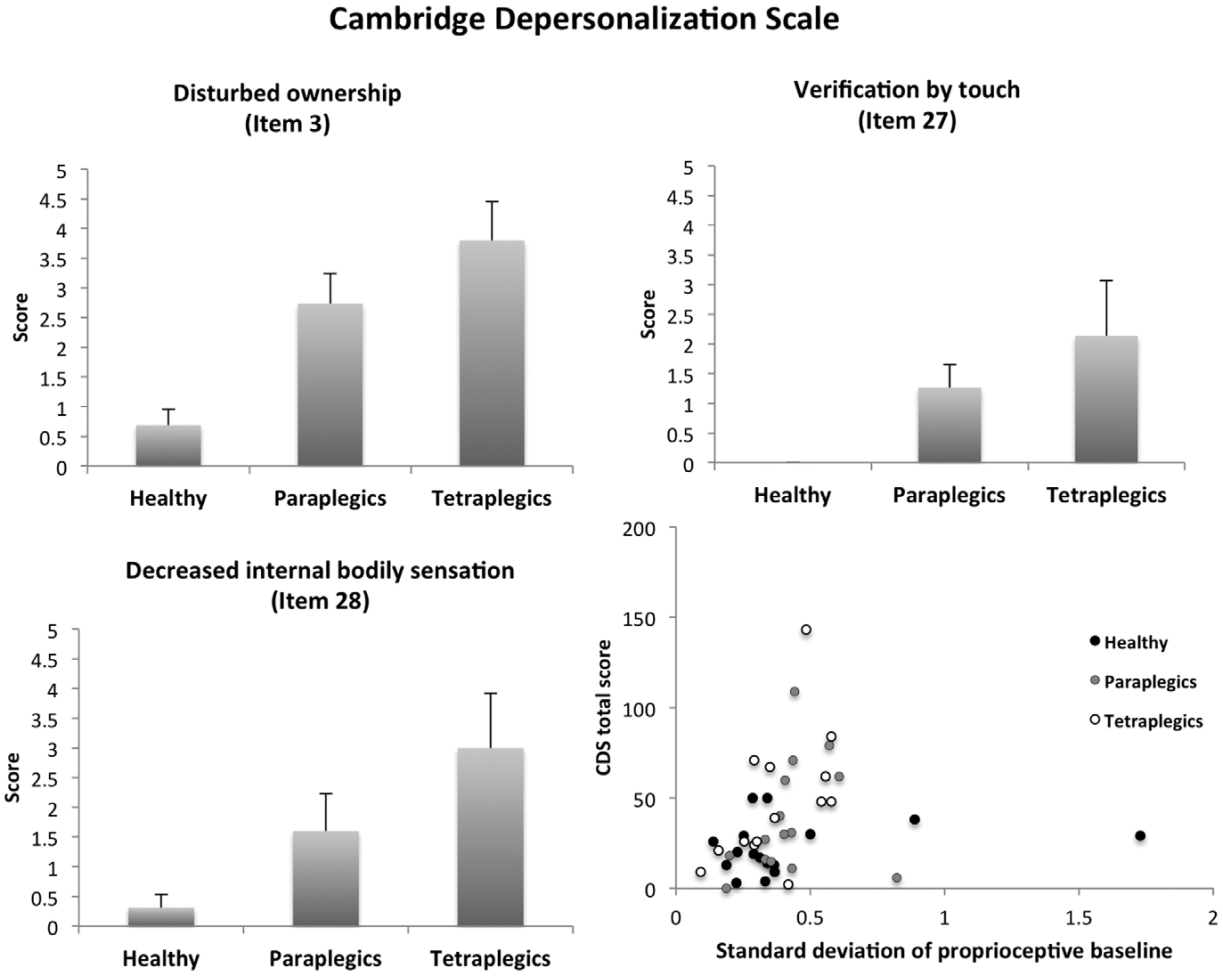
A–C) Cambridge Depersonalization Scale (CDS) items with significant differences between the healthy subjects and the SCI patients A) Item 3 ‘Parts of my body feel as if they don't belong to me’; B) Item 27 ‘I have to touch myself to make sure that I have a body or a real existence’; C) Item 28 ‘I seem to have lost some bodily sensations (e.g. of hunger and thirst) so that when I eat or drink, it feels an automatic routine’. Higher scores indicate increasing agreement with the statement. D) Significant correlation between the standard deviation of the proprioceptive judgment (i.e. accuracy in their baseline judgment) and the total CDS score.

A non-parametric comparison (Mann-Whitney U test) suggested that the total CDS score (the arithmetic sum of all items, including frequency and duration) differed between the SCI patients and healthy participants (z = 1.93, p = 0.05), with the former having higher scores (mean±SEM: 41.1±6.1) than the latter group (22.8±3.6). However, no significant difference in total CDS score was found between patients with paraplegia and those with tetraplegia (z = 0.57, p = 0.5). Further, in order to better characterize which specific aspects of the bodily self are altered in patients, we identified items with maximal between-group variations by comparing the scores for each item between groups separately. Only the following 3 items were found to differ significantly between the healthy subjects and those with SCI: ‘Parts of my body feel as if they don't belong to me’ (z = 3.7, p>0.001; Item 3), ‘I have to touch myself to make sure that I have a body or a real existence’ (z = 2.9, p = 0.004; Item 27), ‘I seem to have lost some bodily sensations (e.g. of hunger and thirst) so that when I eat or drink, it feels like an automatic routine’ (z = 2.3, p = 0.02; Item 28). These three items also showed a group effect when categorized as healthy, paraplegic, and tetraplegic, revealing increasing scores from healthy participants to tetraplegic patients (Pearson’s chi square; Item 3: χ^2^ = 14.6, p = 0.001; Item 27: χ^2^ = 8.3, p = 0.016; Item 28: χ^2^ = 6.2, p = 0.045; Figures 2). Furthermore, Item 3 showed a slight positive correlation between lesion level and CDS score (Spearman correlation; r = 0.36, p = 0.05).

### Baseline as a Measure of Proprioceptive Accuracy

Participants’ judgment regarding index finger position in the absence of stimulation was comparable between the groups as indicated by the 2×3 analysis of variance (ANOVA; judgment pre/post × group) using the average index finger position judged at baseline, which showed no significant group effect (F_(2,42)_ = 0.02, p = 0.89), no difference in pre-and post illusion baseline judgment (F_(1,42)_ = 0.06, p = 0.80), and no significant interaction (F_(2,42)_ = 0.35, p = 0.56). Furthermore a 2×3 ANOVA (pre/post × group) on the standard deviation suggested that there was no difference in accuracy between groups (F_(2,42)_ = 0.23, p = 0.79), no difference between pre- and post-experimental judgment (F_(1,42)_ = 3.1, p = 0.09, slight tendency toward decreased standard deviation after the experiment), and no interaction (F_(2,42)_ = 0.04, p = 0.96). Therefore, errors in hand localization *per se* are unlikely to play any role in the 3 groups. However, while we found comparable accuracy between the groups, correlative analysis suggested an association between standard deviation in the baseline proprioceptive measure (i.e. precision in the task) and CDS score (Spearman’s rho = 0.47, p = 0.001; Figure 2 right bottom).

### Rubber Hand Illusion

To obtain an overview of the distribution of the two different components of the illusion between the groups, four categories were defined according to the presence or absence of the illusion as quantified by the questionnaire and according to the presence or absence of proprioceptive drift. The illusion was considered present (Q+) with a positive score on question 3, ‘It felt as if the rubber hand was my hand’ [19]. Drift was defined as present (D+) when a larger proprioceptive drift was measured in the synchronous condition than in the asynchronous condition (Table 2). Such a drift is thought to be an objective measure of how strongly the rubber hand is integrated into the participant’s body schema and a reliable measure of visual capture of touch and proprioception.

**Table 2:** Overview of the participants grouped according to the presence or absence of subjective (illusory ownership as measured by Q3 in the questionnaire) and objective (proprioceptive drift) measures of rubber hand illusion.

	Category			
Group	Q–D-	Q–D+	Q+D−	Q+D+
**Healthy**	2	2	2	**10**
**Paraplegic**	1	1	**8**	5
**Tetraplegic**	5	2	1	**6**

The category with the highest prevalence in each group is indicated in bold.

Analysis of the contingency table using the chi square test indicated a significant difference among the 3 groups (χ^2^ = 13.5, p = 0.04). The data suggested that healthy participants predominantly experienced a complete illusion. Patients with paraplegia most often experienced an illusion but no proprioceptive drift, while a different pattern was observed in patients with tetraplegia: about half of the patients did feel complete illusion while the other half did not show either drift or illusion.

To better characterize the 3 groups, the proprioceptive drift and subjective report data were analyzed separately. The proprioceptive drift, which was normally distributed (Shapiro-Wilk test for all conditions: p>0.20), was analyzed using a mixed model ANOVA with stroking (synchronous/asynchronous) as a within-subjects factor and subject group (healthy/paraplegic/tetraplegic) as a between-subjects factor. Post-hoc pairwise comparisons were performed using the Sidak test. The questionnaire data were not normally distributed (Shapiro-Wilk test in all conditions: p<0.05); thus, we used a Kruskal–Wallis test for the between-group factor Group and the Wilcoxon test for the within-group factors of Synchrony and Question.

#### Proprioceptive drift

A 2×3 mixed-model ANOVA (synchrony/asynchrony X group) for proprioceptive drift showed a significant main effect of synchrony (F_(1,42)_ = 6.7, p = 0.01) and a significant interaction effect between group and synchrony (F_(1,42)_ = 3.3, p = 0.048). Post-hoc test results indicated that healthy participants showed a typical significantly higher drift during synchronous stroking versus asynchronous stroking (p = 0.003; Sidak comparison), while patients with tetraplegia demonstrated only a trend in the same direction (p = 0.07) and no difference was observed in patients with paraplegia (p = 0.65).

**Figure 3:**
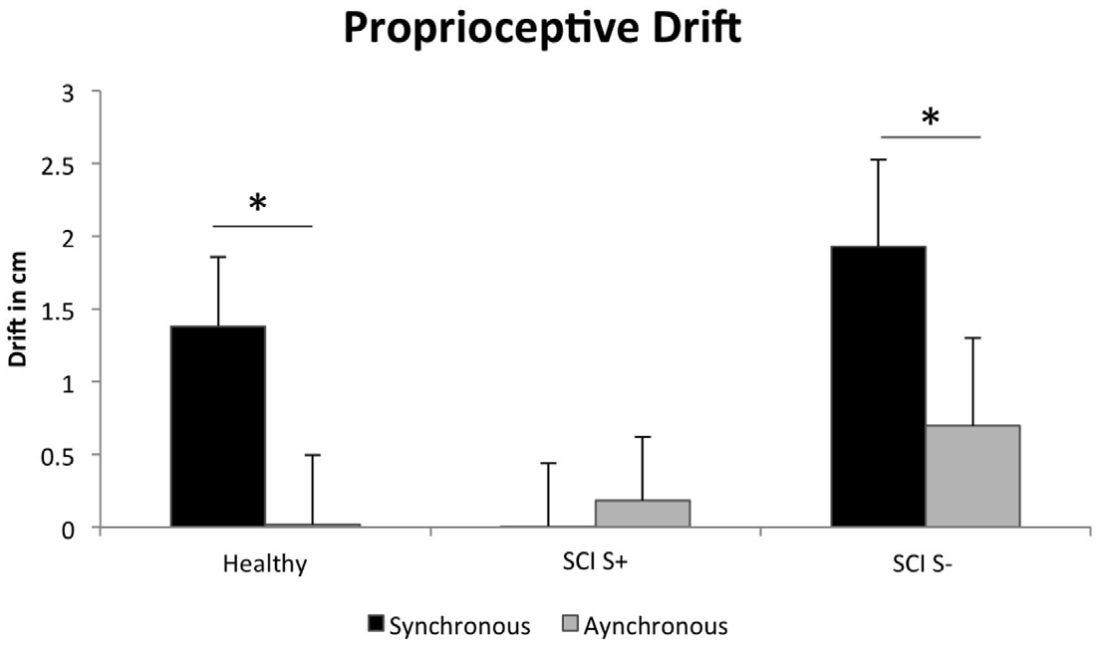
Significant interaction between group (SCI+, SCI-, healthy) and synchrony (synchronous, asynchronous stroking) for proprioceptive drift (mean ± SEM). The values plotted are as compared to the baseline. * p<0.05.

To determine if the reduced tactile sensation in the left hand that characterized tetraplegic patients (see Figure 1) is associated with response to RHI, we regrouped the patients into those with intact tactile sensation in their left hands (S+) and those with reduced tactile sensation (S−). We then conducted the ANOVA (synchrony/asynchronous × group (S+, S−, healthy subjects) again, which indicated a main effect of synchrony (F_(1,42)_ = 9.3, p = 0.004, larger drift after synchronous stroking) as well as an interaction with group (F_(2,42)_ = 4.2, p = 0.02; Figure 3). Post-hoc comparisons show that synchrony influenced proprioceptive drift in healthy (p = 0.003) and in S− patients (p = 0.03) but not in S+ patients (p = 0.40). Further, the drift in the synchronous condition was significantly smaller in the S+ group than in the S− group (p = 0.04). Thus, proprioceptive drift appears linked to sparing of peripheral tactile afferences in SCI patients, tentatively suggesting that reorganization in SCI patients may be driven by tactile-dependent mechanisms.

#### Questionnaire

The descriptive results of the questionnaire data are shown according to the group, synchrony and questions (illusion-relevant versus illusion-irrelevant, see [10]) in Table 3. The Kruskal-Wallis test revealed no significant effect of group (all p values >0.05) on either illusion-relevant or -irrelevant items after either synchronous or asynchronous stroking. However, within each group, an effect of synchrony was evident for the illusion-relevant questions (Wilcoxon Signed-Rank test, all p values <0.01). Furthermore, in all groups, there was a significant difference between the illusion-relevant and -irrelevant questions only after synchronous stroking (all p values <0.01; asynchronous stroking: all p values >0.05). The results thus suggest a strong illusion, as evidenced by the classic RHI questionnaire in all groups, but there seems to be no significant modulation between groups.

**Table 3:** Mean score and standard error for each group in the illusion relevant (average of Q1–Q3) versus the illusion-irrelevant (average of Q4–Q9) items of the rubber hand illusion questionnaire after synchronous and asynchronous stimulation.

Group	Q1–Q3	Wilcoxon test Q1–Q3	Q4–Q9	Wilcoxon test Q1–Q3 vs Q4–Q9	Q1–Q3	Q4–Q9	Wilcoxon test Q1–Q3 vs Q4–Q9
**Healthy**	4.9 (±0.5)	*p = 0.001*	2.8 (±0.3)	*p = 0.001*	1.8 (±0.2)	2(±0.2)	*p = 0.33*
**Paraplegic**	5.5 (±0.5)	*p = 0.001*	2.3 (±0.4)	*p = 0.001*	2 (±0.4)	1.6 (±0.3)	*p = 0.17*
**Tetraplegic**	4.2 (±0.6)	*p = 0.003*	1.9 (±0.3)	*p = 0.003*	1.5 (±0.3)	1.3 (±0.1)	*p = 0.48*

Values above 4 correspond to an affirmation.

#### Correlation between proprioceptive drift and questionnaire

We found a significant non-parametric Spearman’s correlation between drift and illusion-relevant questionnaire scores after synchronous stroking in healthy participants (r = 0.62, p = 0.01), but not in paraplegic (r = 0.41, p = 0.12) or tetraplegic (r = 0.08, p = 0.78) patients. When the results were expressed as relative values to those observed in the asynchronous condition, the correlations between drift and questionnaire data were not significant in any of the groups (all p values >0.05).

## Discussion

In this study, we used a depersonalization questionnaire and the RHI paradigm to investigate changes in 2 different aspects of the sense of the body and of the self in patients with altered peripheral somatosensory and motor processing due to SCI. Three important new findings were obtained: (1) SCI patients showed elevated depersonalization scores. (2) The results obtained using the RHI paradigm suggest that illusory body ownership is not significantly altered in SCI patients compared to healthy participants. In particular, no evidence was found to support the enhancement of visual capture in patients with tetraplegia. (3) Rather surprisingly, in patients with left hand intact tactile sensation, illusory body ownership did not result in a proprioceptive drift as it does in both healthy subjects and patients with reduced tactile sensation.

### Disownership and Feelings of Detachment from One’s Own Body

Increased depersonalization symptoms were observed in SCI patients, especially in those with tetraplegia. It has been postulated that depersonalization symptoms can be attributed to an ‘alteration in the usual mechanism of comparison of immediate sensory perception with memory records’ [5]. Such a mismatch between online sensorimotor processing and the cortical sensorimotor representation of the body (also compare [20]) could have caused the increase in depersonalization symptoms in SCI patients. Similar mismatch mechanisms between actual sensory input and the cortical body representation have been suggested to underlie phantom pain and other phantom sensations, which are commonly observed after SCI [21], [22], [23]. Yet, as there was no correlation between the duration since the lesion occurred and the CDS score, a more plausible alternative explanation is, that a mismatch between online visual and proprioceptive information about the body (i.e. seeing the body but not feeling it), could underlie the elevated scores. A discrepancy between visual and bodily (i.e. vestibular) signals has been suggested to underlie high depersonalization scores in patients with vestibular lesions [8], [9]. It should, however, be noted that the CDS includes various aspects of depersonalization and derealization symptoms ranging from emotional numbness to problems with autobiographical memory [24].

The results described here suggest that SCI patients differ from healthy participants, mainly in 2 subcomponents. First, the SCI patients showed higher detachment from their internal sensations. This finding is in line with the literature that suggests decreased interoceptive sensitivity and awareness in SCI patients, presumably due to autonomic disturbances [25]. Second and more relevant in the context of this study, SCI patients showed higher feeling of detachment from their bodies in that they reported that they often felt as if their bodies did not belong to them, leading them to compulsively touch or pinch their leg with their hand to reassure themselves of their bodily existence. The score concerning disturbed body ownership further correlated with spinal cord lesion height, suggesting that lesions affecting more body segments cause greater disturbance of body ownership. This finding indicated a direct link between the feeling of body ownership and peripheral somatosensory and motor processes and is in line with findings of decreased feeling of body ownership in patients with locked-in syndrome [26]. Our results may suggest that therapies to reinforce a patient’s body ownership should be developed for the treatment of SCI patients.

Independent of the presence of a spinal cord lesion was a correlation between proprioceptive accuracy of the index finger (as measured by the mean deviation of several consecutive localization measurements) and the CDS total score. This correlation suggests that higher CDS score is associated with less precise proprioceptive feedback. The finding could be related to clinical literature suggesting that patients with schizophrenia or related symptoms show deficits in proprioception (e.g. [27]), sensory deprivation studies that related lack of proprioceptive updating to disturbed self processes [7], literature showing that dopaminergic drugs induce both alteration in the perception of the bodily self as well as decreased proprioceptive sensitivity [28], and the theoretical assumption outlining the importance of proprioception in the construction of the sense of self. However, as the task was not designed as a proprioceptive acuity task, these findings are rather explorative and should be taken with caution.

### Illusory Ownership of a Fake Hand

Illusory body part ownership of a fake hand as measured by the RHI questionnaire [10] was not differentially affected among the groups. Even though Table 1 suggests that tetraplegic patients experienced an illusion less often than healthy participants and paraplegic patients as determined by the questionnaire, this could not be evidenced statistically using nonparametrical factor analysis. The data thus showed only a main effect of the synchrony of stroking, revealing a stronger illusory ownership for the rubber hand during synchronous compared to asynchronous stroking in all groups. This is a classic and very robust finding in the RHI paradigm (e.g. [10]) and has been linked to the fact that during synchronous stroking, the brain merges the observed and felt stroking into a single perception. In healthy participants as in SCI patients, this visual capture of touch thus leads to an illusory feeling of ownership and to an illusory sensation of touch indicating that ownership of a fake hand can be induced to a similar extent in patients with body-brain disconnection and in healthy participants. Therefore, multisensory illusions may represent a tool for altering and restoring disturbed feelings of body ownership, subjective sensation of touch, and embodiment in SCI patients. For example, long-term exposure to multisensory illusions, such as embodiment of a virtual avatar or a wheelchair or illusory projection of touch onto body parts with lost/decreased tactile sensation, could help patients to gain alternative embodiment. Similarly, illusions based on visual capture have been successfully used to induce illusory body ownership and decrease neuropathic pain in paraplegic patients [29].

### Ownership of Body Parts Versus General Body Ownership

Interestingly, while the CDS data suggest disturbed body ownership among SCI patients, especially in those with high lesion levels, illusory ownership of a fake hand (as measured by the RHI questionnaire) was not significantly different among the groups. This apparent discrepancy in the results may be explained by the following 3 lines of evidence, which are listed in order of increasing degree of relevance.

First, it is important to account for the differences in scales between the 2 questionnaires. While the scale of ownership used in the RHI questionnaire indicates the strength of the illusion, the frequency and duration of the illusion are captured by the CDS. The 2 measurements are also dissimilar in terms of the time period of reference (the RHI questionnaire asks only about the last 2 minutes, while the CDS addresses a much longer time period) as well as focusing differently on spontaneity (the RHI questionnaire asks about the effects of a specific stimulation, while the CDS inquires regarding spontaneous changes in the self).

Second, we should consider that the RHI questionnaire asks participants about *illusory* ownership of a rubber hand, while the CDS measures changes in *actual* body sensation. Thus, one may speculatively suggest that disturbed actual body ownership among SCI patients might increase their propensity to experience illusory ownership of a fake or alternative body part.

The third (and possibly most important) line of evidence concerns the fact that the RHI questionnaire specifically targets body ownership of the stimulated left hand, while the CDS questionnaire inquires about embodiment and ownership in general. Important differences have been described between global body ownership and body part ownership (see e.g. [30] for a discussion). It could thus be suggested that SCI patients demonstrate disturbed aspects of the global self, while their body part ownership (specifically that of the hand) is still intact.

### Lack of Drift in Patients with Paraplegia

While no difference in the level of illusory ownership was evidenced between groups, evidence for the proprioceptive drift classically found in the RHI (see e.g. [30] for a related discussion) was found in healthy participants and tetraplegic patients, but not in paraplegic patients. At first glance, this group difference might seem surprising because hand somatomotor functions are entirely intact in paraplegic patients and no difference compared to healthy participants would be expected. Furthermore, results of regrouping the patients according to functional level suggest that the same pattern is found in tetraplegic patients with intact somatosensory hand functions. Interestingly, important adaptive processes for the remaining input from the spinal cord in the process of establishing a new body reference have been described in SCI patients. At the functional level, it has been convincingly demonstrated that even loss of sensory input due to massive disconnection of lower limb can induce reorganization of the cortical representation of the finger (e.g. [31]) and hand [4] with a shift towards the somatomotor leg area. At the structural level, studies confirm the presence of reorganization due to expansion of the remaining afferents into deprived cortical (primary sensory cortex; [32]) and subcortical areas [4]. This is followed by a large-scale cortical reorganization, mainly due to reduction of gray matter in areas representing the lower limb [32].

We suggest that these strong changes in somatomotor and higher-level body areas may underlie the lack of a proprioceptive drift, even with a preserved perceptual illusion of ownership for the rubber hand. Neural correlates of the RHI have shown to mainly involve the sensorimotor, premotor, parietal, and insular areas [4]); all areas that may partially be reorganized in patients with paraplegia. Interestingly, a recent PET study in healthy participants [33], [34], [35] found a negative correlation between the proprioceptive drift and neural activity in the contralateral primary and secondary somatosensory cortices. The authors suggested that individuals with a small or negative drift show a strong internal proprioceptive representation of the body that is not captured by vision. Thus, it might be that the enlarged hand representation in the sensorimotor cortex due to cortical reorganization [34] strengthens the internal somatosensory body representation and thus prevents the overwriting of proprioceptive information through visual capture. It should be noted that such a hypothetical increase in the somatosensory representation did not prevent the occurrence of RHI [4] but only prevented the illusion from overwriting proprioceptive information regarding hand localization. This finding is supported by increasing evidence that the subjective experience of illusory ownership and limb recalibration may be two dissociated aspects of the RHI paradigm [35], which can be differentially affected by sensorimotor disorders (see e.g. [36] for a discussion).

Of course, dramatic cortical reorganization has also been observed in tetraplegic patients; however, as the somatomotor functions of the hand are also affected, a small increase in the cortical hand representation in S1 is expected and the face area is expected to extend to the hand area. Nevertheless, as all tetraplegic patients included in this study had at least some residual sensory input from the hand, a similar mechanism as hypothesized for paraplegic patients could have impeded the stronger visual capture and resulted in larger drift in tetraplegic patients as we hypothesized. This is in line with the present study’s finding of a significant correlation between the objective and subjective dimensions of the RHI in healthy subjects but not in SCI patients, suggesting that the integrity of afferent and efferent connections between the body and the brain is necessary in order to fully induce this illusion. Further research, especially neuroimaging studies, will be necessary to directly verify the hypothesis drawn in the current paper.

## References

[1] Deneve S, Pouget A (2004) Bayesian multisensory integration and cross-modal spatial links. Journal of physiology, Paris 98: 249–258. 10.1016/j.jphysparis.2004.03.011 15477036

[2] Berlucchi G, Aglioti SM (2010) The body in the brain revisited. Experimental brain research 200: 25–35. 19690846 10.1007/s00221-009-1970-7

[3] Giummarra MJ, Gibson SJ, Georgiou-Karistianis N, Bradshaw JL (2008) Mechanisms underlying embodiment, disembodiment and loss of embodiment. Neuroscience and biobehavioral reviews 32: 143–160. 17707508 10.1016/j.neubiorev.2007.07.001

[4] Henderson LA, Gustin SM, Macey PM, Wrigley PJ, Siddall PJ (2011) Functional reorganization of the brain in humans following spinal cord injury: evidence for underlying changes in cortical anatomy. The Journal of neuroscience 31: 2630–2637. 21325531 10.1523/JNEUROSCI.2717-10.2011 PMC6623700

[5] Penfield W, Rasmussen T (1950) The Cerebral Cortex of Man: A Clinical Study of Localization of Function. New York: Macmillan.

[6] Simeon D, Guralnik O, Hazlett EA, Spiegel-Cohen J, Hollander E, et al (2000) Feeling unreal: a PET study of depersonalization disorder. The American journal of psychiatry 157: 1782–1788. 11058475 10.1176/appi.ajp.157.11.1782

[7] Gimbel L (1975) The pathology of boredom and sensory deprivation. The Canadian journal of psychiatric nursing 16: 12–13. 1043016

[8] Jauregui-Renaud K, Sang FY, Gresty MA, Green DA, Bronstein AM (2008) Depersonalisation/derealisation symptoms and updating orientation in patients with vestibular disease. Journal of neurology, neurosurgery, and psychiatry 79: 276–283. 10.1136/jnnp.2007.122119 17578858

[9] Sang FY, Jauregui-Renaud K, Green DA, Bronstein AM, Gresty MA (2006) Depersonalisation/derealisation symptoms in vestibular disease. J Neurol Neurosurg Psychiatry 77: 760–766. 16464901 10.1136/jnnp.2005.075473 PMC2077438

[10] Botvinick M, Cohen J (1998) Rubber hands ‘feel’ touch that eyes see. Nature 391: 756. 9486643 10.1038/35784

[11] Cole J (2004) Still lives. Cambridge: MIT press.

[12] Moseley GL, Olthof N, Venema A, Don S, Wijers M, et al (2008) Psychologically induced cooling of a specific body part caused by the illusory ownership of an artificial counterpart. Proceedings of the National Academy of Sciences of the United States of America 105: 13169–13173. 18725630 10.1073/pnas.0803768105 PMC2529116

[13] Kammers MP, Rose K, Haggard P (2011) Feeling numb: temperature, but not thermal pain, modulates feeling of body ownership. Neuropsychologia 49: 1316–1321. 21354190 10.1016/j.neuropsychologia.2011.02.039

[14] Sierra M, Berrios GE (2000) The Cambridge Depersonalization Scale: a new instrument for the measurement of depersonalization. Psychiatry research 93: 153–164. 10725532 10.1016/s0165-1781(00)00100-1

[15] Simeon D, Kozin DS, Segal K, Lerch B, Dujour R, et al (2008) De-constructing depersonalization: further evidence for symptom clusters. Psychiatry research 157: 303–306. 17959254 10.1016/j.psychres.2007.07.007

[16] Sierra M, Baker D, Medford N, David AS (2005) Unpacking the depersonalization syndrome: an exploratory factor analysis on the Cambridge Depersonalization Scale. Psychological medicine 35: 1523–1532. 16164776 10.1017/S0033291705005325

[17] de Vignemont F (2011) Embodiment, ownership and disownership. Consciousness and cognition 20: 82–93. 20943417 10.1016/j.concog.2010.09.004

[18] Lopez C, Lenggenhager B, Blanke O (2010) How vestibular stimulation interacts with illusory hand ownership. Consciousness and cognition 19: 33–47. 20047844 10.1016/j.concog.2009.12.003

[19] Zeller D, Gross C, Bartsch A, Johansen-Berg H, Classen J (2011) Ventral premotor cortex may be required for dynamic changes in the feeling of limb ownership: a lesion study. The Journal of neuroscience 31: 4852–4857. 21451023 10.1523/JNEUROSCI.5154-10.2011 PMC3119817

[20] Carruthers G (2008) Types of body representation and the sense of embodiment. Consciousness and cognition 17: 1302–1316. 18359642 10.1016/j.concog.2008.02.001

[21] Bors E (1951) Phantom limbs of patients with spinal cord injury. AMA archives of neurology and psychiatry 66: 610–631. 14868030 10.1001/archneurpsyc.1951.02320110075007

[22] Burke DC, Woodward SM (1976) Pain and phantom sensation in spinal paralysis. In: Vinken PJ, Bruyn GW, editors. Injuries of the spine and spinal cord; Part II. Amsterdam: North-Holland Publishing Company.

[23] Curt A, Yengue CN, Hilti LM, Brugger P (2011) Supernumerary phantom limbs in spinal cord injury. Spinal Cord 49: 588–595. 21079624 10.1038/sc.2010.143

[24] Simeon D, Abugel J (2006) Feeling unreal: depersonalization disorder and the loss of the self. New York: Oxford University Press, USA.

[25] Montoya P, Schandry R (1994) Emotional experience and heartbeat perception in patients with spinal cord injury and control subjects. Journal of Psychophysiology 8: 289–296.

[26] Nizzi MC, Demertzi A, Gosseries O, Bruno MA, Jouen F, et al. (2011) From armchair to wheelchair: How patients with a locked-in syndrome integrate bodily changes in experienced identity. Consciousness and cognition. 10.1016/j.concog.2011.10.010 22100276

[27] Erwin BJ, Rosenbaum G (1979) Parietal lobe syndrome and schizophrenia: comparison of neuropsychological deficits. Journal of Abnormal Psychology 88: 234–241. 500950 10.1037//0021-843x.88.3.234

[28] Rosenbaum G, Cohen BD, Luby ED, Gottlieb JS, Yelen D (1959) Comparison of sernyl with other drugs: simulation of schizophrenic performance with sernyl, LSD-25, and amobarbital (amytal) sodium; I. Attention, motor function, and proprioception. AMA archives of general psychiatry 1: 651–656. 14438905 10.1001/archpsyc.1959.03590060113013

[29] Moseley GL (2007) Using visual illusion to reduce at-level neuropathic pain in paraplegia. Pain 130: 294–298. 17335974 10.1016/j.pain.2007.01.007

[30] Blanke O, Metzinger T (2009) Full-body illusions and minimal phenomenal selfhood. Trends in cognitive sciences 13: 7–13. 19058991 10.1016/j.tics.2008.10.003

[31] Tsakiris M, Haggard P (2005) The rubber hand illusion revisited: visuotactile integration and self-attribution. J Exp Psychol Hum Percept Perform 31: 80–91. 15709864 10.1037/0096-1523.31.1.80

[32] Bruehlmeier M, Dietz V, Leenders KL, Roelcke U, Missimer J, et al (1998) How does the human brain deal with a spinal cord injury? The European journal of neuroscience 10: 3918–3922. 9875370 10.1046/j.1460-9568.1998.00454.x

[33] Ehrsson HH, Spence C, Passingham RE (2004) That’s my hand! Activity in premotor cortex reflects feeling of ownership of a limb. Science 305: 875–877. 15232072 10.1126/science.1097011

[34] Tsakiris M (2010) My body in the brain: a neurocognitive model of body-ownership. Neuropsychologia 48: 703–712. 19819247 10.1016/j.neuropsychologia.2009.09.034

[35] Tsakiris M, Hesse MD, Boy C, Haggard P, Fink GR (2007) Neural Signatures of Body Ownership: A Sensory Network for Bodily Self-Consciousness. Cereb Cortex 17: 2235–44. 17138596 10.1093/cercor/bhl131

[36] Rohde M, Di Luca M, Ernst MO (2011) The Rubber Hand Illusion: feeling of ownership and proprioceptive drift do not go hand in hand. PloS one 6: e21659. 21738756 10.1371/journal.pone.0021659 PMC3125296

